# Isolation and characterization of mesenchymal stem/stromal cells from *Ctenomys minutus*

**DOI:** 10.1590/1678-4685-GMB-2018-0012

**Published:** 2018-11-29

**Authors:** Mayra Ramos de Jesus Pereira, Valéria Rodrigues Pinhatti, Maiele Dornelles da Silveira, Cristina Araujo Matzenbacher, Thales Renato Ochotorena de Freitas, Juliana da Silva, Melissa Camassola, Nance Beyer Nardi

**Affiliations:** ^1^ Laboratory of Stem Cells and Tissue Engineering, Universidade Luterana do Brasil, Canoas, RS, Brazil; ^2^ Departamento de Genética, Universidade Federal do Rio Grande do Sul, Porto Alegre, RS, Brazil

**Keywords:** Mesenchymal stem/stromal cells, tuco-tuco, Ctenomys minutus

## Abstract

Mesenchymal stem/stromal cells (MSCs) are multipotent cells distributed in all tissues and characterized by adherence, morphology, immunophenotype and trilineage differentiation potential. The present study aimed to isolate and characterize adherent MSC-like populations from different tissues of *Ctenomys minutus*, a threatened wildlife rodent popularly known as tuco-tuco. Adherent cells were isolated from bone marrow, brain, liver, pancreas and adipose tissue of three adult animals collect in southern Brazil. Cultures showed typical morphology and proliferation potential. Adipose-derived MSCs showed trilineage potential. Cultures derived from adipose tissue, bone marrow and brain were immunophenotyped with negative results for CD31, CD44, CD45, CD106, and MHC class II, as well as strong positive results for CD29. Low fluorescence levels were seen for CD49d, CD90.2 and CD117. Cultures were negative for CD49e, except for brain-derived cultures that were weakly positive. CD11b was negative in adipose-derived MSCs, but positive in brain and bone marrow-derived cultures. The scratch assay showed high migration potential for pancreas and adipose tissue-derived cells. This study represents the first report of isolation and characterization of cultures having characteristics of MSCs from *Ctenomys minutus*. The collection of biological information for biobanks represents an important contribution to the creation of strategies for prevention of loss of genetic diversity.

## Introduction

Mesenchymal stem/stromal cells (MSCs) are multipotent cells with a perivascular niche, and therefore distributed in all vascularized tissues in the organism (Meirelles *et al.*, 2006). They are adherent cell populations, characterized as MSCs by their morphology, immunophenotype and trilineage differentiation potential (Dominici *et al.*, 2006). Their therapeutic potential, explained mainly by their capacity to secrete many different bioactive molecules that display anti- apoptotic, angiogenic, anti-scarring, immunomodulatory, and chemoattractant properties (Meirelles *et al.*, 2008), has been explored in animal and human diseases (reviewed by Meirelles *et al.*, 2016).

The idea of establishing biobanks for the purpose of conservation of threatened or endangered species was introduced around 35 years ago (reviewed by Saragusty *et al.*, 2016). A recent review (Machado *et al.*, 2016) described the initiatives to establish biobanks in Brazil, stressing the importance of increasing these studies. Natural or artificial gametes, embryos, induced pluripotent stem cells (iPSCs), and fibroblast cell lines are considered as the most appropriate biomaterial to preserve. MSCs are also very good candidates due to their multipotency, ease to isolate and expand, and potential to originate iPSCs (Zomer *et al.*, 2015). However, in spite of the great number of studies describing MSCs isolated from different tissues of many species of mammals (reviewed by Uder *et al.*, 2017), very few reports describe these cells in wildlife animals.

The Ctenomyidae family includes one genus (*Ctenomys*) comprising approximately 70 species (Bidau, 2015) that show high rates of speciation and karyotype variation (Castillo *et al.*, 2012). Popularly known as tuco-tucos, they are usually solitary and have low mobility, with a distribution typically in small patches of suitable habitats (Kubiak *et al.*, 2017). Many of the species are considered as threatened or endangered by the IUCN Red List of Threatened Species.

The species *C. minutus* is distributed over approximately 500 km along of the coastal plains of southern Brazil (Freitas, 1995; Freygang *et al.*, 2004). They have been used in population genetics studies due to their karyotype variability, which suggests that this species can undergo speciation due to geographical isolation (Freygang *et al.*, 2004). The present study aimed to isolate and characterize adherent cell populations from different tissues of *C. minutus*.

## Material and Methods

### Reagents and culture media

Normal culture medium (NM) was composed of Dulbecco’s modified Eagle’s medium (DMEM) with HEPES (free acid, 2.5–3.7 g/l) and 10% fetal bovine serum (Gibco BRL, Grand Island, NY, USA). Ca^2+^- and Mg^2+^-free Hank’s balanced salt solution (HBSS) was used to wash tissues and cells. All reagents used in this study were from Sigma Chemical Co. (St Louis, MO, USA), unless otherwise stated.

### Sample collection

Two female *Ctenomys minutus* were collected in Mostardas (RS, Brazil) and another one in Jaguaruna (SC, Brazil). The animals were caught live with Oneida Victor No. 0 traps (Oneida Victor, Cleveland, OH, USA) with rubber covers and were transported to the laboratory within 24 h. They were humanely euthanized, and brain, liver, and pancreas were collected. Adipose tissue was obtained from the inguinal region, and bone marrow was collected by flushing the cavity of femurs with normal culture medium. The research protocol was approved by the Ethics Committee on Animal Use of the Universidade Federal do Rio Grande do Sul (Protocol 31925 - CEUA).

### Adherent cells isolation and culture

Bone marrow cells were resuspended in HBSS. Brain, liver, pancreas, and adipose tissue were cut into small pieces, washed and digested with collagenase type I (250 U/mL in DMEM/10 mM HEPES) for 30 min at 37 °C. All tissues were centrifuged at 400 x *g* for 10 min at room temperature. The pellets were resuspended in 3.5 mL NM containing 1% antibiotic-antimycotic solution (GIBCO BRL), seeded in 6-well dishes (3.5 mL/well) and incubated at 37 °C in a humidified atmosphere containing 5% CO_2_. Three days later, the NM was replaced, with removal of non-adherent cells.

For subculture, the adherent monolayer was incubated with 0.25% trypsin and 0.01% EDTA for 5 min, collected, and washed in HBSS. The cultures were split at ratios empirically determined for two subcultures a week at most. Cells were used in passages 3 to 6 in all experiments, except for determination of population doubling times when older cultures were also analyzed.

### Morphological analysis and photographs

Adherent cell cultures were routinely observed with an inverted phase-contrast microscope (Axiovert 25; Carl Zeiss, Hallbergmoos, Germany). Photomicrographs were taken with a digital camera (AxioCam MRc, Carl Zeiss), using AxioVision 3.1 software (Carl Zeiss).

### Culture kinetics

For determination of the proliferation rate, cells were grown to 80–85% confluence and counted at every passage from passage 3 to 8. The number of viable cells was determined using a Neubauer chamber after trypan blue staining. The population doubling time (PDT) of the cultures was calculated by the formula: log(final cell number) - log(initial cell number) = K x T, where K is the generation constant (0.008963) and T is time in days (Roth, 2006).The mean population doubling time of cultures derived from two or three independent donors was assessed in triplicates and expressed in days. PDT for brain-derived cultures was determined for one culture only. Some cultures were followed for extended periods, as detailed below.

### MSC differentiation

Trilineage differentiation was induced by plating MSCs at 10^4^ cells/cm^2^ in 6-well culture plates and maintaining them for up to 8 weeks in inducing media. For osteogenesis, NM was supplemented with 10^–8^ M dexamethasone, 5 μg/mL ascorbic acid 2-phosphate and 10 mM β-glycerophosphate. Adipogenic medium included 10^-8^ M dexamethasone, 2.5 μg/mL insulin, 100 μM indomethacin, and 3.5 μM rosiglitazone. For chondrogenic differentiation, NM was supplemented with 6.25 μg/mL insulin, 10 ng/mL TGF-β1, and 50 nM ascorbic acid 2- phosphate. All media were changed twice a week. Differentiation was observed by washing the cultures, fixing with 4% paraformaldehyde, and staining with Alizarin Red S, Oil Red O, and Alcian Blue, respectively. Experiments were performed in biological triplicates.

### Immunophenotyping

The immunophenotype of MSCs was determined by flow cytometry. The cells were trypsinized, centrifuged, and incubated for 30 min at 4 °C with antibodies conjugated with fluorescein isothiocyanate (FITC), R-phycoerythrin (PE), allophycocyanin (APC) or Alexa Fluor 488 or 700. Since no species-specific antibodies were available, antibodies against mouse or rat antigens (BD Pharmingen, San Diego, CA, USA, or eBioscience, La Jolla, CA, USA) were tested, as presented in Table 1. Excess antibody was removed by washing, and the cells were analyzed on an ACCURI C6 flow cytometer (Becton Dickinson, USA). At least 10,000 events were collected, and the results were analyzed with the BD Accuri C6 software.

**Table 1:** Antibodies used for immunophenotyping *C. minutus* MSC cultures.

Antigen	Target species	Manufacturer	Catalog number
CD11b	Mouse	BD Pharmingen	553311
CD11b	Mouse	eBioscience	53-0112
CD11b/c	Rat	eBioscience	12-0110
CD29	Rat	BD Pharmingen	555005
CD31	Rat	eBioscience	12-0311
CD31	Mouse	eBioscience	50-0310
CD44	Mouse	eBioscience	56-0441
CD45	Mouse	eBioscience	11-0461
CD45	Rat	eBioscience	11-0451
CD49d	Mouse	BD Pharmingen	553157
CD49e	Mouse	BD Pharmingen	557447
CD90.2	Mouse	BD Pharmingen	553006
CD106	Mouse	eBioscience	11-1061
CD117	Mouse	BD Pharmingen	553355
MHC Classe II	Rat	eBioscience	12-0920
MHC Classe II	Mouse	eBioscience	12-5322

### *In vitro* scratch assay

Adherent cells were allowed to grow to 70-80% confluence on 6-well culture plates, when a pipette tip was used to scratch the monolayer (Kramer *et al.*, 2013). Migration of the cells into the scratch area was evaluated by images recorded at 0, 24, 48, and 72 h using an inverted phase-contrast microscope (Axiovert 25, Carl Zeiss). Photomicrographs were analyzed with ImageJ software (National Institutes of Health version 1.48v). Cultures derived from adipose tissue (n = 2) and pancreas (n = 2) were analyzed, and experiments were performed in triplicates.

### Statistical analysis

Results are expressed as mean ± standard deviation. Data were analyzed and graphs were generated using Prism 5 software (GraphPad Software Inc, San Diego, CA, USA). Data were tested for normality and analyzed by one-way analysis of variance followed by Tukey’s post hoc test, with significance set at *p* < 0.05.

## Results

### Isolation and cultivation of adherent cells

**Figure 1:**
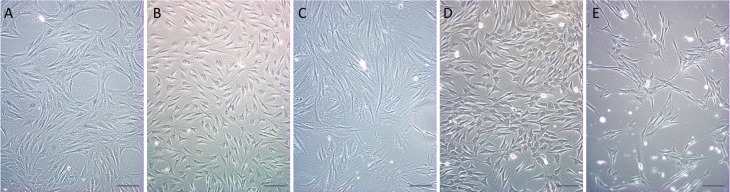
Morphology of cultures. Cultures established from all organs and tissues presented the typical fibroblastoid morphology of mesenchymal stem/stromal cells: (A) brain; (B) adipose tissue; (C) bone marrow; (D) liver; (E) pancreas. Scale bar = 50 μm.

After collagenase digestion (or only cell disaggregation, in the case of bone marrow) and plating, cultures of adherent cells were established from all organs and tissues (Figure 1). Cultures isolated from all tissues were frozen and remain available for future studies.

The cultures showed the typical fibroblastoid morphology of mesenchymal stem/stromal cells, and were maintained until passage 9 or 10, when most of them began to show a decrease in proliferation capacity. Cultures derived from brains (n = 2), however, had a different behavior and showed intense proliferation until passage 20 or 23, when cultures were terminated. Further characterization was performed for adipose tissue-derived MSC cultures, with additional analyses of other cultures, as presented in Table 2.

**Table 2:** *C. minutus* tissues from which adherent cultures were established and characteristics analyzed or assays performed. The results are described in the text.

Tissue	Morphology	Proliferation	Differentiation potential	Immunophenotype	Scratchassay
Brain	Yes	Yes	No	Yes	No
Liver	Yes	No	No	No	No
Pancreas	Yes	Yes	No	No	Yes
BM	Yes	Yes	No	Yes	No
Adipose	Yes	Yes	Yes	Yes	Yes

### Proliferation

**Figure 2:**
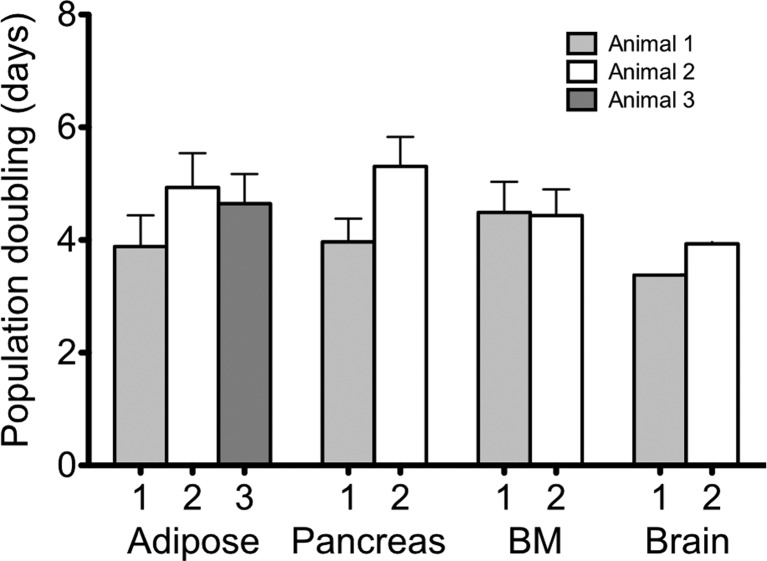
Mean values of population doublings. Viable cells were counted at each passage (P3 to P8) of cultures derived from adipose tissue (n = 3), pancreas (n = 2), bone marrow (BM, n = 2) and brain (n = 2), for determination of population doubling times.

Population doubling times of cultures derived from adipose tissue (n = 3), pancreas (n = 2), bone marrow (n = 2), and brain (n = 2) were determined by counting viable cells at each passage (P3 to P8). As presented in Figure 2, no significant differences were observed among cultures from the different organs and tissues. Although brain-derived cultures could be cultured for much longer periods, only a slightly lower PDT was observed between passages 3 and 8.

### MSC differentiation

**Figure 3:**
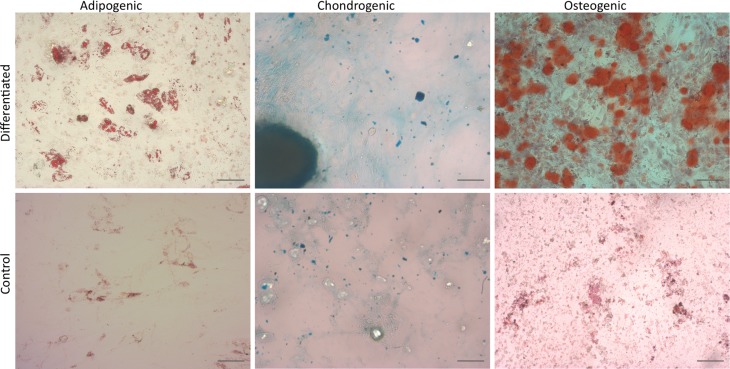
Differentiation potential of adipose-derived MSCs. Representative results show trilineage differentiation potential of cultures. Scale bar = 50 μm.

The differentiation potential of *C. minutus* MSCs was analyzed only for adipose tissue-derived cultures. As represented in Figure 3, the cells showed trilineage potential.

### Immunophenotyping

**Figure 4:**
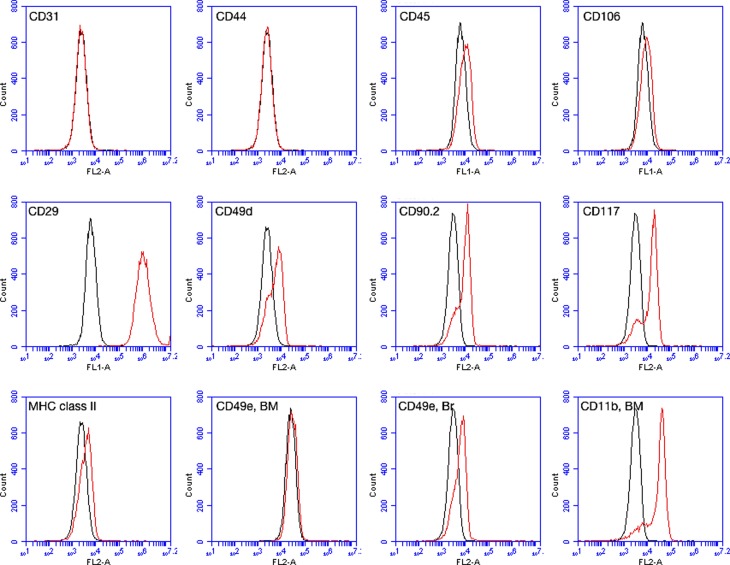
Immunophenotype of adipose-derived (n = 3) and brain- and bone marrow (BM)-derived (n = 2) MSCs. Representative results show that all cultures were negative for CD31, CD44, CD45, CD106, and MHC class II. Cultures were strongly positive for CD29 and weakly positive for CD49d, CD90.2, and CD117. Adipose- and BM-derived MSCs were negative for CD49e, and brain (Br)-derived cells were weakly positive. Two of the anti-CD11b antibodies tested showed positive marking for Br- and BM-derived cultures.

MSCs derived from adipose tissue of the three samples were immunophenotyped, with additional analysis of bone marrow- and brain-derived cultures from two donors. A range of antibodies specific for mouse or rat antigens was tested, as shown in Table 1. Representative results are presented in Figure 4. All cultures were negative for surface markers CD31, CD44, CD45, CD106, and MHC class II, and they were also strongly positive for CD29. All cultures showed positive results also for CD49d, CD90.2, and CD117, but with lower levels of fluorescence. These results suggest some heterogeneity among cells in the cultures, since a negative population seems also be present. Cultures were negative for CD49e, except for brain-derived cultures that were weakly positive.

The three anti-CD11b antibodies tested gave different results. One of them (mouse-specific) had consistently negative results (not shown), while the other two (one anti-mouse and the other anti-rat) showed negative results for adipose-derived MSCs (not shown), but positive labeling of brain-derived (not shown) and bone marrow-derived cultures.

### *In vitro* scratch assay

**Figure 5:**
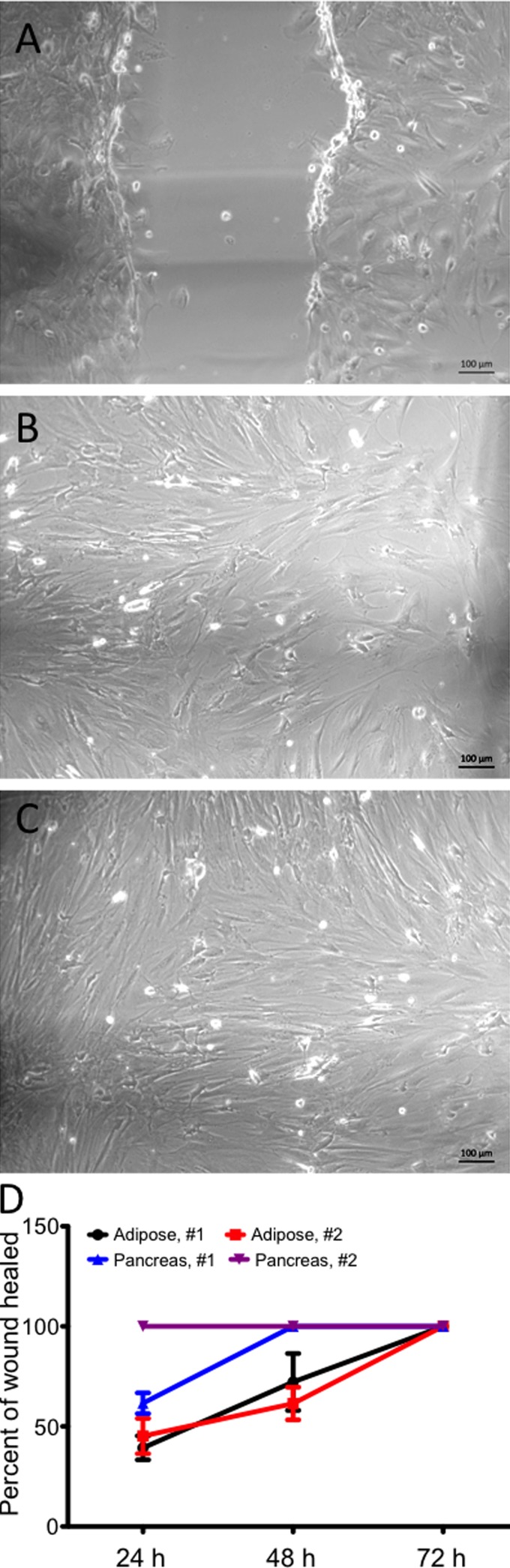
*In vitro* scratch assay. Migration of cells into a scratch produced in adipose tissue and pancreas-derived cultures from two donors (#1 and #2) was evaluated by images recorded at 0, 24, 48, and 72 h. The percent of closed scratch (or “healed wound”) was determined with ImageJ software. Values are mean ± standard deviation of three independent experiments.

To evaluate the capacity of adherent cells to migrate *in vitro*, a scratch assay was performed with pancreas and adipose tissue-derived cultures, and the closure of the scratch area was recorded at 24, 48, and 72 h (Figure 5). Although adipose-derived cells showed a high capacity of migration, with total covering of the cell-free area within 72 h, migration of cells from the pancreas-derived cultured was much faster. For cells of the first donor, total closure was seen at 48 h, and with cells of donor two, the scratch area was closed at 24 h.

## Discussion

Adherent cell cultures characterized in this study were isolated from different tissues: bone marrow, adipose tissue, brain, liver, and pancreas. The results confirm the existence of cell populations having characteristics of MSCs in various body tissues, as shown for many different species (Meirelles *et al.*, 2006, 2008), also for *C. minutus*.

Cells derived from all the tissues presented the fibroblastoid morphology that characterizes MSCs (Pittenger *et al.*, 1999). Their proliferation potential was evaluated by determining the population doubling time, with similar values seen for all cultures. This method has been used, with similar results, for cultures isolated from human adipose tissue (Markarian *et al.*, 2014), and for comparing human MSCs isolated from adipose tissue, bone marrow, and umbilical cord blood (Baksh *et al.*, 2003; Kern *et al.*, 2006). Cultures derived from umbilical cord blood showed significantly greater PDT than other tissues, for which results were similar to the present study. In addition, these populations were analyzed separately according to passage, showing that the PDT increases proportionally to the age of the culture. Similar results were described for canine adipose-derived MSCs (Lee *et al.*, 2014).

As one of the most important criteria to define human and animal MSCs (reviewed by Uder *et al.*, 2017), *C. minutus* adipose tissue-derived cultures showed trilineage differentiation potential. Some studies quantify the differentiation potential of MSCs isolated from different tissues. As shown by Meirelles *et al.* (2006) and others, the potential for differentiation is related to the tissue of origin of MSCs. In the present study, there was no comparison of cultures with respect to this criterion.

Since no species-specific antibodies were available for *C. minutus* and murine antibodies were used, the results must be seen with caution. Negative results were observed for most of the antigens considered as negative in MSCs (Dominici *et al.*, 2006), including CD31, CD45, CD106, and MHC Class II. However, for CD11b, which is also absent in MSCs of other species, the results were not so clear, with positive results for two of the antibodies in brain and bone marrow-derived cultures. CD44, a positive marker of MSCs, was also negative in all cultures tested.

CD29 (Integrin β1), one of the positive markers of MSCs (Uder *et al.*, 2017), was the most consistently positive antigen in all cultures. For the other markers, CD49d, CD90.2, and CD117, most cells presented a positive profile but with lower fluorescence intensity. The results seem to indicate the presence of a small population of negative cells. For CD49e, also considered a MSC marker (Mafi *et al.*, 2011), only brain-derived cultures showed a weakly positive result.

In addition to the immunophenotype patterns established by the International Society for Cellular Therapy for human MSCs isolated from bone marrow (Dominici *et al.*, 2006) and adipose tissue (Bourin *et al.*, 2013), a large number of studies have investigated the surface profile of MSCs from other tissues in various species (reviewed by Mafi *et al.*, 2011; Mildmay-White and Khan, 2017; Uder *et al.*, 2017). Although limited by the lack of species specificity, the present results are in accordance with the literature reports. The positive results seen for CD11b are possibly due to cross-reactivity with other surface antigen(s) present in MSCs of this species. Additional studies are needed to confirm the level of positivity of the markers CD49d, CD90.2, and CD117, as well as to determine whether the negative results for CD49e are real, or due to structural differences that do not allow recognition by the antibody used.

Cultures derived from adipose tissue, and particularly pancreas, showed high migration capacity. Similar results have been found in other studies. In a similar assay, Arora *et al.* (2015) observed that human MSCs isolated from Wharton’s jelly show rapid cell migration, with closure of the scratch area within 22 hours. MSCs isolated from human adipose tissue closed 35% of the artificial injury within 30 hours, but the addition of VEGF_165_ or PDGF-BB increased this effect for 65% and 93%, respectively (Amos *et al.*, 2008).

Our results show for the first time that cells with the characteristic MSC features can be isolated from different tissues of *C. minutus*. These results add to the small number of studies that have investigated this type of multipotential cell in wildlife species. MSCs with similar characteristics have been isolated from regenerating antlers of fallow deer (*Dama dama*, Rolf *et al.*, 2008), from adipose tissue of the brown bear (*Ursus arctos*, Fink *et al.*, 2011), from bone marrow of the collared peccary (*Tayassu tajacu*, Argôlo Neto *et al.*, 2016), from adipose tissue, horn and skin of the marsh deer (*Blastocerus dichotomus*, Rola LD, 2017, PhD thesis, Faculdade de Ciências Agrarárias e Veterinárias, Universidade Estadual Paulista, Brazil), and from the skin of three wildlife rodents in the Amazon region in Brazil (*Oecomys concolor*, *Hylaeamys megacephalus,* and *Proechimys roberti*, Rissino JD, 2012, MSc thesis, Universidade Federal do Pará, Brazil). The results were also similar to those of the present study, and the cultures demonstrated fibroblastoid morphology, high proliferation *in vitro,* and trilineage differentiation potential. In addition, Machado *et al.* (2017) described the isolation and preservation of fibroblasts from crab-eating fox (*Cerdocyon thous*).

This study represents the first report of the isolation and characterization of cultures having characteristics of mesenchymal stromal/stem populations from *Ctenomys minutus*. The preservation of frozen samples represents a valuable tool for further studies. Considering the ecological vulnerability of this genus, the collection of biological information for the creation of biobanks represents an important contribution to the creation of strategies for the preservation of species and prevention of loss of genetic diversity.

## References

[B1] Amos PJ, Shang H, Bailey AM, Taylor A, Katz AJ, Peirce SM (2008). IFATS collection: The role of human adipose-derived stromal cells in inflammatory microvascular remodeling and evidence of a perivascular phenotype. Stem Cells.

[B2] Argôlo NM, Feitosa MLT, Silva SS, Fernandes PB, Pessoa GT, Bezerra DO, Almeida HM, Carvalho YKP, Rocha AR, Silva LMC, Carvalho MAM (2016). Isolation, expansion, differentiation and growth kinetics essay in mesenchymal stem cells culture from the bone marrow of collared peccaries (*Tayassu tajacu*). Acta Scient Veter.

[B3] Arora S, Saha S, Roy S, Das M, Jana SS, Ta M (2015). Role of nonmuscle myosin II in migration of Wharton’s jelly-derived mesenchymal stem cells. Stem Cells Dev.

[B4] Baksh D, Davies JE, Zandstra PW (2003). Adult human bone marrow-derived mesenchymal progenitor cells are capable of adhesion-independent survival and expansion. Exp Hematol.

[B5] Bidau CJ, Patton JL, Pardinas UFJ, D’Elia G (2015). Family Ctenomyidae Lesson, 1842. Mammals of South America.

[B6] Bourin P, Bunnell BA, Casteilla L, Dominici M, Katz AJ, March KL, Redl H, Rubin JP, Yoshimura K, Gimble JM (2013). Stromal cells from the adipose tissue-derived stromal vascular fraction and culture expanded adipose tissue-derived stromal/stem cells: A joint statement of the International Federation for Adipose Therapeutics and Science (IFATS) and the International Society for Cellular Therapy (ISCT). Cytotherapy.

[B7] Castilho CS, Gava A, Freitas TRO (2012). A hybrid zone of the genus *Ctenomys*: A case study in southern Brazil. Genet Mol Biol.

[B8] Meirelles LS, Chagastelles PC, Nardi NB (2006). Mesenchymal stem cells reside in virtually all post-natal organs and tissues. J Cell Sci.

[B9] Meirelles LS, Caplan AI, Nardi NB (2008). In search of the *in vivo* identity of mesenchymal stem cells. Stem Cells.

[B10] Meirelles LS, Bellagamba BC, Camassola M, Nardi NB (2016). Mesenchymal stem cells and their relationship to pericytes. Front Biosci.

[B11] Dominici M, Le Blanc K, Mueller E, Slaper-Cortenbach I, Marini F, Krause D, Deans R, Keating A, Prockop D, Horwitz E (2006). Minimal criteria for defining multipotent mesenchymal stromal cells. Cytotherapy.

[B12] Fink T, Rasmussen JG, Emmersen J, Pilgaard L, Fahlman Å, Brunberg S, Josefsson J, Arnemo JM, Zachar V, Swenson JE, Fröbert O (2011). Adipose-derived stem cells from the brown bear (*Ursus arctos*) spontaneously undergo chondrogenic and osteogenic differentiation *in vitro*. Stem Cell Res.

[B13] Freitas TRO (1995). Geographic distribution and conservation of four species of the genus *Ctenomys* in southern Brasil. Stud Neotrop Fauna Environ.

[B14] Freygang CC, Marinho JR, Freitas TRO (2004). New karyotypes and some considerations of *Ctenomys minutus* (Rodentia: Ctenomidae) on the coastal plain of the Brazilian state of Rio Grande do Sul. Genetica.

[B15] Kern S, Eichler H, Stoeve J, Klüter H, Bieback K (2006). Comparative analysis of mesenchymal stem cells from bone marrow, umbilical cord blood, or adipose tissue. Stem Cells.

[B16] Kubiak BB, Galiano D, Freitas TR (2017). Can the environment influence species home-range size? A case study on *Ctenomys minutus* (Rodentia, Ctenomyidae). J Zool.

[B17] Kramer N, Walzl A, Unger C, Rosner M, Krupitza G, Hengstschläger M, Dolznig H (2013). In vitro cell migration and invasion assays. Mutat Res.

[B18] Lee KS, Kang HW, Lee HT, Kim HJ, Kim CL, Song JY, Lee KW, Cha SH (2014). Sequential sub-passage decreases the differentiation potential of canine adipose-derived mesenchymal stem cells. Res Vet Sci.

[B19] Machado LC, Oliveira VC, Paraventi MD, Cardoso RNR, Martins DS, Ambrósio CE (2016). Maintenance of Brazilian biodiversity by germplasm bank. Pesq Vet Bras.

[B20] Machado LC, Roballo KCS, Cury FS, Ambrósio CE (2017). Female reproductive system morphology of crab-eating fox (*Cerdocyon thous*) and cryopreservation of genetic material for animal germplasm bank enrichment. Anat Histol Embryol.

[B21] Mafi P, Hindocha S, Mafi R, Griffin M, Khan WS (2011). Adult mesenchymal stem cells and cell surface characterization - a systematic review of the literature. Open Orthop J.

[B22] Markarian CF, Frey GZ, Silveira MD, Chem EM, Milani AR, Ely PB, Horn AP, Nardi NB, Camassola M (2014). Isolation of adipose-derived stem cells: A comparison among different methods. Biotechnol Lett.

[B23] Mildmay-White A, Khan W (2017). Cell surface markers on adipose-derived stem cells: A systematic review. Curr Stem Cell Res Ther.

[B24] Pittenger MF, Mackay AM, Beck SC, Jaiswal RK, Douglas R, Mosca JD, Moorman MA, Simonetti DW, Craig S, Marshak DR (1999). Multilineage potential of adult human mesenchymal stem cells. Science.

[B25] Rolf HJ, Kierdorf U, Kierdorf H, Schulz J, Seymour N, Schliephake H, Napp J, Niebert S, Wölfel H, Wiese K (2008). Localization and characterization of STRO-1 cells in the deer pedicle and regenerating antler. PLoS One.

[B26] Saragusty J, Diecke S, Drukker M, Durrant B, Ben-Nun FI, Galli C, Göritz F, Hayashi K, Hermes R, Holtze S (2016). Rewinding the process of mammalian extinction. Zoo Biol.

[B27] Uder C, Brückner S, Winkler S, Tautenhahn HM, Christ B (2017). Mammalian MSC from selected species: Features and applications. Cytometry A.

[B28] Zomer HD, Vidane AS, Gonçalves NN, Ambrósio CE (2015). Mesenchymal and induced pluripotent stem cells: General insights and clinical perspectives. Stem Cells Cloning.

